# Supplementary material for: Ovarian Extracellular Matrix Mechanics Regulate Oocyte‐Follicle Interactions During Female Reproductive Aging
Source: Aging Cell. 2026 Aug 13;25(8):e70668. doi: 10.1111/acel.70668 (PMC13471645; doi:10.1111/acel.70668)
Supplement: Supplementary file 1 [file ACEL-25-e70668-s001.docx]

**Supplementary Figures for:**

**Ovarian extracellular matrix mechanics regulate oocyte-follicle interactions during female reproductive aging**

Xingyu Shen^1^, Haiyang Wang^1,2^, Gefei Cao^1^, Yaelim Lee^1^, Jin Zhu^1^, Loo Chien Wang^3^, Tianyun Zhao^4^, Siok Ghee Ler^4^, Radoslaw M. Sobota^4^, Rong Li^1,5*^, Jennifer L. Young^1,6*^

^1^Mechanobiology Institute (MBI), National University of Singapore, 5A Engineering Drive 1, 117411, Singapore

^2^Institute of Modern Biology, Nanjing University, Nanjing 210008, China

^3^Bioprocessing Technology Institute, 20 Biopolis Way, #02-01 Centros, 138668, Singapore

^4^Institute of Molecular and Cell Biology (IMCB), Agency for Science, Technology and Research (A*STAR), 61 Biopolis Drive, 138673, Singapore

^5^Department of Biological Sciences, National University of Singapore, 16 Science Drive 4, 117558, Singapore

^6^Department of Biomedical Engineering, College of Design and Engineering, National University of Singapore, 4 Engineering Drive 3, 117583, Singapore

*Correspondence: biejly@nus.edu.sg, mbihead@nus.edu.sg

**Supplementary Figures**

Figure S1

**Fig. S1 Mechanical measurements on follicles and ovarian stroma.** (a) Follicle stiffness was measured using atomic force microscopy (AFM). Individual follicles were isolated from fresh ovaries (young and aged mice) and immobilized within a PDMS microwell array prior to AFM indentation. (b) Correlation of ovarian stroma ECM to stiffness was carried out by sectioning fresh ovaries and live-labeling with CNA35 (green) to visualize collagen, followed by stiffness measurements using a nanoindenter. Captured fluorescent images of collagen were correlated to the regions of indentation.

Figure S2

**Fig. S2 | Cell proliferation declines in aged ovarian tissue.** (a) Immunostaining of Ki-67 (yellow) and DAPI (cyan) in ovarian tissue cryosections from young (6 weeks) and aged (14 months) mice. (b) Quantification of cell proliferation, shown as the percentage of Ki-67 positive granulosa cells / follicle in young and aged groups. Data are presented as mean ± SD. n = 20 follicles in the young group and n = 9 follicles in the aged group. (b) teal and pink dots are individual follicles; black circles represent biological replicates. Statistical significance was determined by unpaired two-tailed Student’s t-test. Scale bar: 200 µm.

Figure S3

**Fig. S3 | Differential gene expression of granulosa cells and oocytes cultured in soft versus stiff hydrogels.** (a) After young follicles were cultured for 6 days in soft or stiff hydrogels, granulosa cells and oocytes were separately isolated for bulk RNA sequencing (RNA-seq). (b-e) Represent data from granulosa cell RNA-seq, while (f-g) represents oocyte RNA-seq. (b) Volcano plot showing DEGs in granulosa cells cultured in stiff vs. soft hydrogels with selected mechanoregulated genes highlighted. (c) Heatmap illustrating the hierarchical clustering of DEGs in granulosa cells from follicle culture in soft and stiff hydrogels. (d, e) PPI analysis of DEGs of GCs from follicles cultured in stiff versus soft hydrogels identified the network ‘cell adhesion mediated by integrin’ and “response to mechanical stimulus’. For oocytes, (f) mitochondrial-related genes are highlighted in the volcano plot of DEGs. (g) GSEA of top enriched pathways in stiff versus soft hydrogels showing. Graph shows Normalized Enrichment Scores (NES), with bubble size representing gene count and color indicating statistical significance (-Log_10_Q).

Figure S4

**Fig. S4 | Comparison of *in vivo* (aged vs. young) and *in vitro* (stiff vs. soft) transcriptomes in granulosa cells and oocytes.** (a) Log2 fold-change concordance scatter plot comparing *in vivo* aged vs. young granulosa cells (x-axis) and *in vitro* stiff vs. soft hydrogel-cultured granulosa cells (y-axis). Genes are categorized as significant in both conditions (red), significant in aged vs. young only (blue), significant in stiff vs. soft only (orange), or not significant in either (grey). Pearson correlation coefficient r = 0.108. (b) GSEA plot of enriched Gene Ontology biological process terms in aged vs. young granulosa cells. Bubble size represents the number of genes in each term; color indicates the normalized enrichment score (NES). (c) Log2 fold-change concordance scatter plot comparing *in vivo* aged vs. young oocytes (x-axis) and *in vitro* stiff vs. soft hydrogel-cultured oocytes (y-axis), using the same color categorization as in (a). Pearson correlation coefficient r = 0.034. (d) GSEA plot of enriched Gene Ontology biological process terms in aged vs. young oocytes.

Figure S5

**Fig. S5 | Hippo pathway analysis in granulosa cell RNA-seq data.** (a) Heatmap shows Hippo pathway gene expression of granulosa cells isolated from follicles cultured in soft and stiff hydrogels. Asterisk (*) represents significance. At the protein level, (b) YAP (yellow) immunostaining staining in young follicles cultured in soft and stiff hydrogels was carried out with corresponding (c) nuclear (DAPI, blue) translocation quantification (nuclear to cytoplasmic fluorescence intensity ratio: N/C). (c) teal and pink dots are individual follicles; black circles represent biological replicates. Data is shown as mean ± SD and analyzed using unpaired t-test. Sample size is: 23 follicles from 3 independent experiments in both soft and stiff. Scale bar: 30 µm.

Figure S6

**Fig. S6 | *In vivo* Smad2/3 localization of small and large follicles in ovarian tissue.** (a) SMAD2/3 (yellow) and DAPI (blue) in young mice as a function of follicle size (follicle diameter **⌀** <180 µm - top versus **⌀** >180 µm - bottom). (b) Nuclear translocation of Smad2/3 in granulosa cells was quantified as a function of follicle diameter (nuclear to cytoplasmic fluorescence intensity ratio: N/C). (c) Actin immunostaining shows TZPs from (follicle diameter **⌀** <180 µm - top versus **⌀** >180 µm - bottom) in young mice. (d) Schematic illustration defining the quantified parameters: oocyte diameter, the number of oocyte-adjacent granulosa cells (GCs), and TZPs. (e, f) Quantification of the TZP number per oocyte, TZP number per 10 µm, oocyte diameter, and oocyte-adjacent granulosa cell number versus follicle diameter. (g) TZP density as a function of follicle diameter was quantified. (b, g) teal and pink dots are individual follicles; black circles represent biological replicates. Data in (b) and (g) are presented as mean ± SD. Sample size is: (b) n = 11 (D < 180) and 7 (D > 180) follicles from 4 mice; (e-g) n = 25 (D < 180) and 18 (D > 180) follicles from 3 young mice. Statistical significance was determined by unpaired two-tailed Student’s t-test. Scale bar: 80 µm in (a) and 10 µm in (c).

Figure S7

**Fig. S7 | Mitochondrial membrane potential is reduced in aged oocytes.** Mitochondrial membrane potential was assessed through (a) TMRM (top) and MTG (bottom) in freshly isolated young and aged oocytes. (b) Mitochondrial potential (TMRM/MTG ratio) was quantified, showing a reduction with age. b) teal and pink dots are individual oocytes; black circles represent biological replicates. Data is shown as mean ± SD and analyzed using unpaired t-test. Sample size : n = 22 (Young) and 25 (Aged) oocytes from 6 mice in each group. Scale bar: 20 µm.
